# On the Accuracy and Parallelism of GPGPU-Powered Incremental Clustering Algorithms

**DOI:** 10.1155/2017/2519782

**Published:** 2017-10-11

**Authors:** Chunlei Chen, Li He, Huixiang Zhang, Hao Zheng, Lei Wang

**Affiliations:** ^1^School of Computer Engineering, Weifang University, Weifang, Shandong 261061, China; ^2^School of Electromechanical Engineering, Guangdong University of Technology, Guangzhou 510006, China; ^3^School of Automation, Northwestern Polytechnical University, Xi'an, China; ^4^School of Electrical and Computer Engineering, Georgia Institute of Technology, Atlanta, GA, USA

## Abstract

Incremental clustering algorithms play a vital role in various applications such as massive data analysis and real-time data processing. Typical application scenarios of incremental clustering raise high demand on computing power of the hardware platform. Parallel computing is a common solution to meet this demand. Moreover, General Purpose Graphic Processing Unit (GPGPU) is a promising parallel computing device. Nevertheless, the incremental clustering algorithm is facing a dilemma between clustering accuracy and parallelism when they are powered by GPGPU. We formally analyzed the cause of this dilemma. First, we formalized concepts relevant to incremental clustering like evolving granularity. Second, we formally proved two theorems. The first theorem proves the relation between clustering accuracy and evolving granularity. Additionally, this theorem analyzes the upper and lower bounds of different-to-same mis-affiliation. Fewer occurrences of such mis-affiliation mean higher accuracy. The second theorem reveals the relation between parallelism and evolving granularity. Smaller work-depth means superior parallelism. Through the proofs, we conclude that accuracy of an incremental clustering algorithm is negatively related to evolving granularity while parallelism is positively related to the granularity. Thus the contradictory relations cause the dilemma. Finally, we validated the relations through a demo algorithm. Experiment results verified theoretical conclusions.

## 1. Introduction

### 1.1. Background

Due to the exciting advancements in digital sensors, advanced computing, communication, and massive storage, tremendous amounts of data are being produced constantly in the modern world. The continuously growing data definitely imply great business value. However, data are useless by themselves; analytical solutions are demanded to pull meaningful insight from the data, such that effective decisions can be achieved. Clustering is an indispensable and fundamental data analysis method. The traditional clustering algorithm is executed in a batch-mode; namely, all data points are necessarily loaded into memory of the host machine. In addition, every data point can be accessed unlimited times during the algorithm execution. Nevertheless, the batch-mode clustering algorithm can not adjust the clustering result in an evolving manner. For instance, it is necessary to incrementally cluster the evolving temporal data such that the underlying structure can be detected [1]. In stream data mining, a preprocessing task like data reduction needs the support of incremental clustering [2, 3]. In addition, incremental clustering can significantly contribute to massive data searching [4]. To sum up, the evolving capability of incremental clustering is indispensable under certain scenarios, such as memory-limited applications, time-limited applications, and redundancy detection. A practical application may possess arbitrary combination of the three aforementioned characteristics. The incremental clustering algorithm proceeds in an evolving manner, namely, processing the input data step by step. In each step, the algorithm receives a newly arrived subset of the input and obtains the new knowledge of this subset. Afterwards, historic clusters of the previous step are updated with the new knowledge. Subsequently, the updated clusters serve as input of the next step. With regard to the first step, there is no updating operation. The new knowledge obtained in the first step serves as input of the second step. Application scenarios of incremental clustering generally raise high requirements on computing capacity of the hardware platform. General Purpose Graphic Processing Unit (GPGPU) is a promising parallel computing device. GPGPU has vast development prospects due to following superiorities: much rapider growing computing power than CPU, high efficiency-cost ratio, and usability.

### 1.2. Motivation and Related Works

Our previous work revealed that the existing incremental clustering algorithms are confronted with an accuracy-parallelism dilemma [5, 6]. In this predicament, the governing factor is the evolving granularity of the incremental clustering algorithm. For instance, the point-wise algorithms proceed in fine granularity. In each step, the algorithms only receive a single data point (serving as the new knowledge); this point will either be assigned to an existing historic cluster or induce an independent cluster in the historic clusters. Such algorithms generally achieve favorable clustering accuracy. However, they sacrifice parallelism due to strong data dependency. Namely, the next data point cannot be processed until the current one is completely processed. Modern GPGPUs can contain thousands of processing cores, while the number of existing historic clusters needs to increase progressively even if the number eventually reach the magnitude of thousand. GPGPU can fully leverage its computing power only if it runs abundant threads in a SIMD (Single Instruction Multiple Data) manner (commonly twice the number of processing cores or even more). In addition, the work-depth is inevitably no less than the amount of input data points under point-wise setting. Consequently, the computing power is ineluctably underutilized. Moreover, more kernel launches (GPGPU code execution) are required if work-depth is larger. Time overhead of kernel launch is generally high. Some representative point-wise incremental clustering algorithms were elaborated in [7–19]. Ganti et al. used block-evolving pattern to detect changes in stream data [20]. Song et al. adopted the block-wise pattern and proposed an incremental clustering algorithm of GMM (Gaussian Mixture Model) [21]. The algorithm of [21] proceeds in coarse granularity. Each step contains three substeps. First, obtain the new knowledge by running the standard EM (Expectation Maximum) algorithm on a newly received data block. Second, identify the statistically equivalent cluster pairs between the historic clusters and the new clusters. Finally, merge the equivalent cluster pairs separately. The standard EM algorithm of GMM is inherently GPGPU-friendly [22]. The algorithm of [21] maintains the inherent parallelism. However, its clustering accuracy exhibits degradation by order of magnitude, compared to its batch-mode counterpart (the standard EM algorithm of GMM) [5]. Moreover, we qualitatively analyzed the reason why block-wise pattern tends to induce accuracy degradation in our previous work [6]. Algorithms of [23, 24] are also block-wise. D-Stream is ostensibly point-wise [25]. Nevertheless, D-Stream is essentially block-wise due to the fact that mapping data points into grids can be parallelized in a SIMD manner. As far as we know, most existing works focus on clustering accuracy. However, existing algorithms, even the block-wise ones, do not explicitly consider algorithm parallelism on SIMD many-core processing devices like GPGPU. Some recent works formally analyzed issues of clustering or machine learning algorithms. Ackerman and Dasgupta pointed out a limitation that incremental clustering cannot detect certain types of cluster structure [26]. They formally analyzed the cause of this limitation and proposed conquering this limitation by allowing extra clusters. Our work is similar to that of [26] in the sense that we also formally analyzed the reason why incremental clustering is inefficient under certain conditions. In contrast, we elaborated our work under the context of GPGPU-acceleration. Ackerman and Moore also formally analyzed perturbation robustness of batch-mode clustering algorithm [27, 28]. Nevertheless, works of [27, 28] only concentrated on classical batch-mode clustering algorithms. Gepperth and Hammer qualitatively analyzed challenges that incremental learning is facing [29]. They pointed out a dilemma between stability and plasticity. However, we focus on the dilemma between accuracy and parallelism.

### 1.3. Main Contribution

In this paper, we extend our previous works of [6, 30] in the following ways. First, some vital concepts (such as incremental clustering and evolving granularity) are formally defined. Second, we formally proved how evolving granularity exerts influence on accuracy and parallelism of incremental clustering algorithms. In this way, the starting point of our previous works can be formally validated. Finally, we demonstrated the theoretical conclusions through a demo algorithm. The conclusions will be the footstone of our future work.

## 2. Formal Definition of Terminologies

### 2.1. Terminologies on Incremental Clustering

**Definition 1 (incremental clustering).**

**x**_1_, **x**_2_, **x**_3_,…, **x**_*T*_ is a series of data points (**x**_*i*_ ∈ *R* ^*d*^, 1 ≤ *i* ≤ *T*). The data points are partitioned into *T*1 sets: **X**_1_, **X**_2_, **X**_3_,…, **X**_*T*1_. This partition satisfied the following conditions:

1. **X**_*j*_ ≠ *∅*  (*j* = 1,2, 3,…, *T*1).
2. If **x**_*i*1_ ∈ **X**_*j*1_, **x**_*i*2_ ∈ **X**_*j*2_  (*i*1 ≠ *i*2, *j*1 ≠ *j*2), then *j*2 > *j*1⇔*i*2 > *i*1.

A data analysis task adopts discrete time system and time stamps are labeled as 1,2, 3,….* This task is incremental clustering if and only if the following applies:*

1. When *t* = 1, the task receives **X**_1_. In time interval [1,2], the task partitions **X**_1_ into clusters and **C**_1_ is the set of these clusters. The entire input to the task is **X**_1_.
2. When *t* = *j*  (*j* = 2,3, 4,…), the task receives **X**_*t*_. In time interval [*t*, *t* + 1], the task resolves the set of clusters **C**_*t*_ such that ∀**x**_*j*_ ∈ ⋃_1≤*k*≤*t*_ **X**_*k*_ can find its affiliated cluster in **C**_*t*_. Entire inputs to the task are **X**_*t*_ and **C**_*t*_.

**Definition 2 (the *t*th step and historic clustering result of the *t*th step).**

An algorithm for incremental clustering is an incremental clustering algorithm. The time interval [*t*, *t* + 1]  (*t* = 1,2, 3,…) is the *t*th step of an incremental clustering algorithm (or step *t* of an incremental clustering algorithm). **C**_*t*_ is the historic clustering result of the *t*th step.

**Definition 3 (micro-cluster).**

Let* batchAlgorithm* represent a batch-mode clustering algorithm. $\vec{\xi }=\left(\xi^{\left(1\right)},\xi^{\left(2\right)},\ldots ,\xi^{\left(p\right)}\right)\in {\left(R^{+}\right)}^{p}\;\;1\leq p\leq d$ is the parameter of* batchAlgorithm*.* batchAlgorithm* can partition **X**_*t*_ into subsets Sub_*k*_  (*k* = 1,2, 3,…, *K*_*t*,new_). $\vec{\xi_{0}}=(\xi_{0}^{\left(1\right)},\xi_{0}^{\left(2\right)},\ldots ,\xi_{0}^{\left(p\right)})\in (R^{+}{)}^{p}$ is a constant vector. Sub_*k*_ is a $\vec{\xi_{0}}$-micro-cluster produced by *batchAlgorithm* if and only if the following applies:

1. **X**_*t*_ = ⋃_1≤*k*≤*K*_*t*,new__Sub_*k*_.
2. ∀*k*1 ≠ *k*2, Sub_*k*1_∩Sub_*k*2_ = *∅*.
3. Sub_*k*_ forms a data cloud in *d*-dimensional space. The hypervolume of this data cloud is positively related to *ξ*_0_^(*u*)^  (*u* = 1,2, 3,…, *p*). Sub_*k*_ contains and only contains one data point if $\vec{\xi_{0}}=\vec{0}$.
4. *ξ*_0_^(*u*)^  (*u* = 1,2, 3,…, *p*) are all preset constant values.

**Definition 4 (batch-mode part and incremental part of step *t*).**

Some incremental clustering algorithms divide step *t* (*t* = 1,2, 3,…) into two parts [21, 23–25]: In the first part, **X**_*t*_ is partitioned into* new clusters (or new micro-clusters)* pursuant to certain similarity metrics. **C**_*t*,new_ is the set of these clusters (or micro-clusters); in the second part, **C**_*t*_ is resolved based on **C**_*t*,new_ and **C**_*t*−1_ (if any). The number of clusters (or micro-clusters) in **C**_*t*,new_ is denoted as *K*_*t*,new_. The first part can be accomplished by a batch-mode clustering algorithm; this part is* the batch-mode part of step t*. The second part is* the incremental part of step t*.

**Definition 5 (benchmark algorithm, benchmark clustering result, and benchmark cluster).**

Denote **X**_1_, **X**_2_, **X**_3_,…, **X**_*T*1_ (pursuant to Definition 1) as *StrParti*. Incremental clustering is applied to *StrParti*, and **C**_*T*0_ is the historic clustering result of the *T*0th step (*T*0 ≤ *T*1); let **X**_*B*_ = ⋃_1≤*k*≤*T*0_ **X**_*k*_. If **X**_*B*_ could be entirely loaded into memory of the host machine and were processed by a certain batch-mode clustering algorithm, then the resulting clusters were in a set of clusters denoted as **C**_*T*0,benchmark_. The batch-mode algorithm is called* benchmark algorithm of the incremental clustering algorithm.* **C**_*T*0,benchmark_ is the benchmark clustering result up to step *T*0. An arbitrary cluster of **C**_*T*0,benchmark_ is a* benchmark cluster*.

**Definition 6 (local benchmark cluster).**

**C**_*T*,Benchmark_ = {Clu_*k*_∣*k* = 1,2, 3,…, *K*_*T*,Benchmark_} is the benchmark clustering result up to the *T*th step; Clu_*k*_  (*k* = 1,2, 3,…, *K*_*T*,Benchmark_) represent the benchmark clusters in **C**_*T*,benchmark_. **X**_*t*_ is the newly received data set of step *t*. All data points of **X**_*t*_ are labeled such that points with the same label are affiliated to the same benchmark cluster. Partition **X**_*t*_ into nonempty subsets, noted as SubClu_*k*_  (*k* = 1,2, 3,…, *K*_*t*_′). These subsets satisfy the following conditions:

1. **X**_*t*_ = ⋃_1≤*k*≤*K*_*t*_′_SubClu_*k*_ and ∀*k*1 ≠ *k*2, SubClu_*k*1_∩SubClu_*k*2_ = *∅*.
2. If **x**_*i*_, **x**_*j*_ ∈ **X**_*t*_ then **x**_*i*_, **x**_*j*_ ∈ SubClu_*u*_⇔**x**_*i*_ and **x**_*i*_ possess the same label.
3. SubClu_*u*_, SubClu_*v*_ ⊂ **X**_*t*_; if **x**_*i*_ ∈ SubClu_*u*_, **x**_*j*_ ∈ SubClu_*v*_  (*u* ≠ *v*), then **x**_*i*_ and **x**_*j*_ have different labels.

SubClu_*k*_  (*k* = 1,2, 3,…, *K*_*t*_′) are called the* local benchmark clusters of step t* or* local benchmark clusters* for short. We abbreviate local benchmark cluster to LBC.

Definitions 1–4 actually provide terminologies to formally interpret the concept of incremental clustering as well as execution mechanism of incremental clustering. Definitions 5 and 6 furnish a benchmark to evaluate the accuracy of an incremental clustering algorithm.

### 2.2. Terminologies on Evolving Granularity, Clustering Accuracy, and Parallelism

**Definition 7 (containing hypersurface of a data set).**

Let Clu_*w*_ ⊂ *R* ^*d*^ represent a set of data points. HS is a hypersurface in the *d*-dimensional space. HS is the* containing hypersurface* of Clu_*w*_, if and only if HS is a close hypersurface and an arbitrary point of Clu_*w*_ is within the interior of HS.

**Definition 8 (envelope hypersurface, envelop body, and envelop hypervolume of a data set).**

Let Clu_*w*_ ⊂ *R* ^*d*^ represent a set of data points. *S*_HS_ = {HS_*u*_∣*u* = 1,2, 3,…, *U*} is the set of containing hypersurfaces of Clu_*w*_. Let **V**_*u*_ represent the hypervolume encapsulated by HS_*u*_. Let HS_*v*_ be a hypersurface. HS_*v*_ is the envelope hypersurface of Clu_*w*_, if and only if HS_*v*_ = arg min_HS_*u*_∈*S*_HS__ **V**_*u*_. Let EN_*w*_ represent the* envelope hypersurface* of Clu_*w*_; the region encapsulated by EN_*w*_ is the envelope body of Clu_*w*_; the hypervolume of this envelope body is the* envelope hypervolume* of Clu_*w*_.

**Definition 9 (core hypersphere, margin hypersphere, core hypervolume, and margin hypervolume of a data set).**

Let Clu_*w*_ ⊂ *R* ^*d*^ be a data set. EN_*w*_ is the envelope hypersurface of Clu_*w*_; **c** **e** **n** **t** **e** **r**_*w*_ represents the geometric center of the envelope body of Clu_*w*_. dist(**x**) represents the distance between **c** **e** **n** **t** **e** **r**_*w*_ and an arbitrary point on EN_*w*_. dist_*w*,min_ = min_**x**∈EN_*w*__dist(**x**); dist_*w*,max_ = max_**x**∈EN_*w*__dist(**x**).

A hypersphere is the* core hypersphere* of Clu_*w*_ if and only if this hypershpere is centered at **c** **e** **n** **t** **e** **r**_*w*_ and its radius is dist_*w*,min_, noted as SPSur_min_. The hypervolume encapsulated by SPSur_min_ is the* core hypervolume* of Clu_*w*_, noted as SP_min_(Clu_*w*_).

A hypersphere is the margin hypersphere of Clu_*w*_ if and only if it is centered at **c** **e** **n** **t** **e** **r**_*w*_ and its radius is dist_*w*,max_, noted as SPSur_max_. The hypervolume encapsulated by SPSur_max_ is the margin hypervolume of Clu_*w*_, noted as SP_max_(Clu_*w*_).

**Definition 10 (core evolving granularity, margin evolving granularity, and average evolving granularity).**

In the *t*th step, the incremental clustering algorithm receives data set **X**_*t*_. **X**_*t*_ is partitioned into nonempty subsets pursuant to certain metrics: Sub_*k*_  (*k* = 1,2, 3,…, *K*_*t*,new_) such that ∀*k*1 ≠ *k*2, Sub_*k*1_∩Sub_*k*2_ = *∅*, and **X**_*t*_ = ⋃_1≤*k*≤*K*_*t*,new__Sub_*k*_. Let SP_min_(Sub_*k*_) be the core hypervolume of Sub_*k*_. Then* in the tth step, the core evolving granularity* of the algorithm is SubGra_min,*t*_ = min_1≤*k*≤*K*_*t*,new__SP_min_(Sub_*k*_).

*Up to the Tth step, the core evolving granularity* of the algorithm is Gra_min,*T*_ = min_1≤*t*≤*T*_SubGra_min,*t*_.

Let SP_max_(Sub_*k*_) be the margin hypervolume of Sub_*k*_. Then* in the tth step, the margin evolving granularity* of the algorithm is SubGra_max,*t*_ = max_1≤*k*≤*K*_*t*,new__SP_max_(Sub_*k*_).

*Up to the Tth step, the margin evolving granularity* of the algorithm is Gra_max,*T*_ = max_1≤*t*≤*T*_SubGra_max,*t*_. Let EN_*k*_ be the envelope hypersurface of Sub_*k*_, and SubSet_*k*_ = EN_*k*_∩Sub_*k*_; |SubSet_*k*_| is the number of data points within SubSet_*k*_; **c** **e** **n** **t** **e** **r**_*w*_ represents the geometric center of the envelope body of Sub_*k*_. dist_*k*,*u*_ represents the distance between **c** **e** **n** **t** **e** **r**_*w*_ and **x**_*u*_. Let ave_dist_*k*_ = (∑_**x**_*u*_∈SubSet_*k*__dist_*k*,*u*_)/|SubSet_*k*_|.

SP_ave_(Sub_*k*_) is the hypervolume of the hypersphere whose center is at **c** **e** **n** **t** **e** **r**_*w*_ and radius is ave_dist_*k*_.* In the tth step, the average evolving granularity* of the algorithm is SP_ave_(Sub_*k*_).

*Up to the Tth step, the average evolving granularity* of the algorithm is Gra_ave,*T*_ = (∑_*t*=1_^*T*^Gra_ave,*t*_ *K*_*t*,new_)/∑_*t*=1_^*T*^ *K*_*t*,new_.

**Definition 11 (different-to-same mis-affiliation).**

Different-to-same mis-affiliation is the phenomenon that, in step *t*, data points from different benchmark clusters are affiliated to the same cluster of **C**_*t*,new_ or **C**_*t*_.

**Definition 12 (same-to-different mis-affiliation).**

Same-to-different mis-affiliation is the phenomenon that, in step *t*, data points from the same benchmark cluster are affiliated to the different clusters of **C**_*t*,new_ or **C**_*t*_.

We adopt Rand Index [31] to measure clustering accuracy. Larger Rand Index means higher clustering accuracy.

There are numerous criterions of cluster separation measurement in the existing literatures. We select Rand Index due to the fact that this criterion directly reflects our intent: measuring the clustering accuracy by counting occurrences of data point mis-affiliations.

**Definition 13 (serial shrink rate (SSR)).**

Let incAlgorithm represent an incremental clustering algorithm. In step *t*  (*t* = 1,2, 3,…), the batch-mode part generates micro_*t*_ micro-clusters. Suppose incAlgorithm totally clustered *N* data points up to step *T*_0_. Up to step *T*_0_, the serial shrink rate of incAlgorithm is

(1) $$\begin{array}{c} SSR=\frac{\left(\sum_{t=1}^{T_{0}}{micro}_{t}\right)}{N}. \end{array}$$

Lower SSR means that less computation inevitably runs in a non-GPGPU-friendly manner. Consequently, smaller SSR means improved parallelism. Work-depth [32] of the algorithm can shrink if SSR is smaller. Hence, more computation can be parallelized.

## 3. Theorems of Evolving Granularity

### 3.1. Further Explanation on the Motivation

GPGPU-accelerated incremental clustering algorithms are facing a dilemma between clustering accuracy and parallelism. We endeavor to explain the cause of this dilemma through formal proofs. The purpose of our explanation is that the formal proofs reveal a possible solution to seek balance between accuracy and parallelism. This basic idea of this solution is discussed as follows.

In the batch-mode part of the *t*th step, data points from different local benchmark clusters may be mis-affiliated to the same micro-cluster of **C**_*t*,new_.  Theorem 14 points out that the upper and lower bounds of the mis-affiliation probability are negatively related to margin evolving granularity and core evolving granularity, respectively. The proof of this theorem demonstrates that larger evolving granularity results in more occurrences of different-to-same mis-affiliation.

The batch-mode part should evolve in fine granularity to produce as many homogeneous micro-clusters as possible. Only in this context, the operations of incremental part are sensible. Namely, the incremental part cannot eliminate different-to-same mis-affiliation induced by the batch-mode part. The incremental part absorbs advantages of point-wise algorithms by processing micro-clusters sequentially. This part should endeavor to avoid both same-to-different and different-to-same mis-affiliations on the micro-cluster-level.

Nevertheless, Theorem 15 proves that parallelism is positively related to evolving granularity. Thus, the contrary relations cause the dilemma.

However, we can adopt GPGPU-friendly batch-mode clustering algorithm in the batch-mode part. Moreover, the total number of micro-clusters is much smaller than that of data points up to a certain step. Consequently, the work-depth can be dramatically smaller than that of a point-wise incremental clustering algorithm.

### 3.2. Theorem of Different-to-Same Mis-Affiliation

**Theorem 14 .**

Let *P*_*t*,mis_ represent the probability of different-to-same mis-affiliations induced by the batch-mode part of the *t*th step. Gra_min,*T*_ and Gra_max,*T*_ are the core evolving granularity and margin evolving granularity up to the *T*th step, respectively. The upper bound of *P*_*t*,mis_ is negatively related to Gra_max,*T*_ and the lower bound of *P*_*t*,mis_ is negatively related to Gra_min,*T*_.

Suppose **X**_*t*_ contains *K*_*t*_′ local benchmark clusters (LBC). *LBCSet*_*t*_ is a set containing these LBCs. Between any two adjacent LBCs there exists a boundary curve segment. The boundary curve segment between LBC *SubClu*_*k*1_ and LBC SubClu_*k*2_  (*k*1, *k*2 = 1,2, 3,…, *K*_*t*_′; *k*1 ≠ *k*2) is noted as Cur^(*k*1,*k*2)^. Obviously, Cur^(*k*1,*k*2)^ and Cur^(*k*2,*k*1)^ represent the same curve segment. We define a probability function to represent *P*_*t*,mis_:

(2) Pt,misr=∑SubCluk1,SubCluk2∈LBCSett,k1<k2curVrCurk1,k2∑SubCluk∈LBCSet,VSubCluk,

where

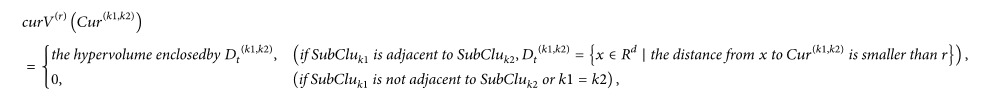
(3)and *V*(SubClu_*k*_) is the hypervolume enclosed by envelope surface of *SubClu*_*k*_.

*P*_*t*,mis_(*r*) reaches the upper bound if *r* equals the radius corresponding to the margin evolving granularity (Definition 10) in step *t*; *P*_*t*,mis_(*r*) reaches the lower bound if *r* equals the radius corresponding to the core evolving granularity (Definition 10) in step *t*.

**Proof**

*In order to more intuitively interpret this theorem, we discuss the upper and lower bounds in two-dimensional space. The following proof can be generalized to higher-dimensional space*.

*In two-dimensional space, the envelop hypersurface (Definition 8) degenerates to an envelope curve. The core hypersphere and margin hypersphere (Definition 9) degenerate to a core circle and a margin circle, respectively. The envelop body degenerates to the region enclosed by the envelope curve. The envelope hypervolume degenerates to the area enclosed by the envelope curve*. Let incAlgorithm represent an incremental clustering algorithm. Each step of incAlgorithm includes batch-mode part and incremental part.

(*1) Partition Data Points Pursuant to Local Benchmark Clusters*. incAlgorithm receives **X**_*t*_ in the *t*th step. Partition **X**_*t*_ into local benchmark clusters (Definition 6, LBC for short): SubClu_*u*_  (*u* = 1,2, 3,…, *K*_*t*_′). Let AR = ⋃_*u*=1_^*K*_*t*_′^SubClu_*u*_. EN_*u*_ is the envelope curve of SubClu_*u*_. *V*(SubClu_*u*_) represents the area enclosed by SubClu_*u*_. Assume that SubClu_*u*_  (*u* = 1,2, 3,…, *K*_*t*_′) are convex sets. (We can partition SubClu_*u*_ into a set of convex sets if it is not a convex set.)

(*2) Partition the Boundary Curve between Two Local Benchmark Clusters into Convex Curve Segments.* Let **C** **u** **r** be the set of boundary curves between any two adjacent LBCs. **C** **u** **r** = {Cur_*k*_∣*k* = 1,2, 3,…, *K*} where Cur_*k*_ is the boundary curve segment between two adjacent LBCs. Consider an arbitrary LBC SubClu_*u*_. Suppose that there are totally *U* LBCs adjacent to SubClu_*u*_. The boundary curves segments are Cur_*u*_^(1)^, Cur_*u*_^(2)^,…, Cur_*u*_^(*U*)^. These boundary curve segments can be consecutively connected to form a closed curve such that only data points of SubClu_*u*_ are within the enclosed region. Further partition Cur_*k*_ into a set of curve segments such that Cur_*k*,*g*_  (*g* = 1,2, 3,…, *G*_*k*_) are all convex curve segments. Cur_*k*,*g*_ can be viewed as a set of data points.

**Figure 1:**
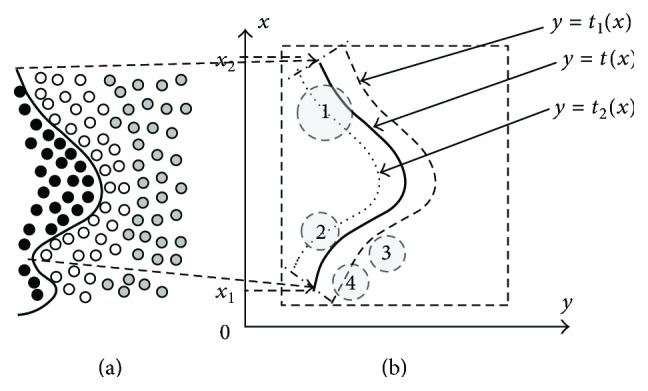
Relation between evolving granularity and different-to-same mis-affiliation induced by the batch-mode part.

*(3) Construct Auxiliary Curves.* Figure 1(a) illustrates an example of adjacent LBCs and a boundary curve. The black, gray, and white small circles represent three distinct LBCs (For clarity of the figure, we use small circles to represent data points). The black bold curve is the boundary curve between the black and white LBCs. We cut out a convex curve segment from this boundary curve, noted as Cur_*k*,*g*_. Figure 1(b) magnifies Cur_*k*,*g*_. Assume that the analysis formula of Cur_*k*,*g*_ is *y* = *t*(*x*)  (*x* ∈ *D*_*k*,*g*_ = [*x*_1_, *x*_2_]).

Let Cir_*E*_ be a circle centered at point *E*. The radius of Cir_*E*_ is* threshold *(*threshold* ∈ *R* ^+^). Place Cir_*E*_ to the right of Cur_*k*,*g*_. Roll Cir_*E*_ along Cur_*k*,*g*_ such that Cir_*E*_ is always tangent to Cir_*E*_. Let Cir_*I*_ be a circle centered at point *I*. The radius of Cir_*I*_ is also* threshold*. Place Cir_*I*_ to the left of Cur_*k*,*g*_. Roll Cir_*I*_ along Cur_*k*,*g*_ such that all points of Cir_*I*_ are always to the left of Cur_*k*,*g*_, except the points of tangency between Cir_*I*_ and Cur_*k*,*g*_. The trajectories of points *E*, *I* form two curves, *y* = *t*_1_(*x*) and *y* = *t*_2_(*x*), respectively. Adjust the starting and ending points of *t*1(*x*) and *t*2(*x*) such that the definition domains of both curves are *D*_*k*,*g*_ = [*x*_1_, *x*_2_].

*(4) Characteristics of New Clusters.* In step *t*, **X**_*t*_ is partitioned into new clusters (or new micro-clusters) (Definition 3) pursuant to certain methods. Let GR be a set containing data points of an arbitrary new cluster. Without loss of generality, let envelope curve of GR be a circle centered at **c** **e** **n** **t** **e** **r**_GR_, noted as ENGR. The radius of this circle is noted as* radius*. We can view **c** **e** **n** **t** **e** **r**_GR_ as a random variable. This random variable represents the possible coordinates of GR's center. Let *D*(SubClu_*u*_) represent a set of all vectors enclosed by envelope curve of SubClu_*u*_ in *d*-dimensional (*d* = 2) space (including vectors on the envelope curve). Let *D*(AR) = ⋃_*u*=1_^*K*_*t*_′^ *D*(SubClu_*u*_). The statistical characteristic of **X**_*t*_ is unknown before **X**_*t*_ is processed. Consequently, it is reasonable to assume that center_GR_ obeys the uniform distribution on *D*(AR). Namely, we assume that every point within *D*(AR) is possible to be GR's center and the probabilities of every point are equal.

*(5) Criterion of Different-to-Same Mis-Affiliation.* Assume that we can neglect distance between the boundary curve and the right side of the black LBC's envelope curve in Figure 1. Similarly, assume that we can neglect distance between the boundary curve and the left side of the white LBC's envelope curve. The criterion of different-to-same mis-affiliation is as follows.

If ENGR∩Cur_*k*,*g*_ ≠ *∅*, then GR contains data points of at least two distinct local benchmark clusters.

Smaller distance between **c** **e** **n** **t** **e** **r**_GR_ and Cur_*k*,*g*_ means higher probability of different-to-same mis-affiliation induced by the batch-mode part; the larger the radius is, the higher the probability is.

In Figure 1(b), two lines (*x* = *x*_1_, *x* = *x*_2_) and two curves (*y* = *t*_1_(*x*), *y* = *t*_2_(*x*)) form an open domain, noted as *D*_threshold_ ∈ *R* ^2^. Let Set represent the set of threshold values that make the following causal relationship hold:

**c** **e** **n** **t** **e** **r**_GR_ ∈ *D*_threshold_ and *threshold* ≤ *radius*⇒ENGR∩Cur_*k*,*g*_ ≠ *∅*.

Part (3) of this proof explained the meaning of* threshold *(*threshold* ∈ *R* ^+^). GR's* threshold with regard to *Cur_*k*,*g*_ is *threshlod*_max_ = max_*threshold*∈Set_ *threshold*. *D*_critical_ = *D*_threshold_|_threshold=threshlod_max__. Different-to-same mis-affiliation can still occur as long as* radius* is sufficiently large even if center_GR_ ∉ *D*_critical_.

**Figure 2:**
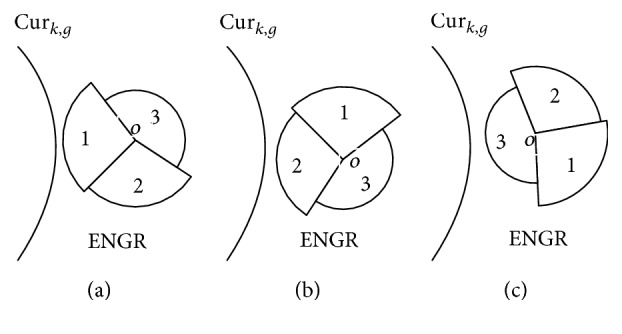
Typical examples of different-to-same mis-affiliations induced by batch-mode part.

*(6) Three Typical Situations of Different-to-Same Mis-Affiliation Induced by Batch-Mode Part.* The value of *threshold*_max_ is dramatically influenced by the following factors: first: shape of LBC's envelop curve and second: the relative positions of Cur_*k*,*g*_ and GR. Shape of LBC's envelope curve is generally irregular. We simplify the proof without loss of generality. As illustrated by Figure 2, assume that three sectors are consecutively connected to form the envelope curve. In addition, three sectors' centers overlapped on point *O*. Radiuses of sectors 1, 2, and 3 are *r*1, *r*2, and *r*3, respectively. *r*1 equals to the radius of the margin envelope circle. *r*3 equals to the radius of the core envelope circle. *r*2 is between *r*1 and *r*3. Generally, distances between point *O* and points on the envelope curve are between *r*1 and *r*3. Sector 2 can represent these ordinary points.

Let GR rotate around *O*. Figures 2(a), 2(b), and 2(c) illustrate three characteristic positions of GR during the rotation. In Figure 2(a), threshold is *r*_1_. *D*_critical_ covers the largest area. In Figure 2(c), threshold is *r*_3_. *D*_critical_ covers the smallest area.

*(7) Probability of Different-to-Same Mis-Affiliation Induced by Batch-Mode Part: Lower Bound.* As aforemetioned, a boundary curve between two LBCs can be partitioned into curve segments. Data points on both sides of a curve segment are affiliated to the same cluster if different-to-same mis-affiliation occurs (this mis-affiliation occurs in the batch-mode part of a certain step). Let Cur_*k*,*g*_ represent a curve segment from a boundary curve. Let *P*_*k*,*g*_ be the probability that data points on both sides of Cur_*k*,*g*_ are affiliated to the same cluster. Considering all boundary curves in **X**_*t*_, *P*_*t*,mis_ represents the total probability of different-to-same mis-affiliation in the batch-mode part of step *t*.

Let *r*_min,*T*_ be the radius of the hypersphere corresponding to core evolving granularity up to step *T*. Assume that auxiliary curves *y* = *t*_1_(*x*) and *y* = *t*_2_(*x*) are constructed under *threshold* = *r*_min,*T*_. On the basis of the previous parts of proof, we can draw the following inequalities:

(4) Pk,g≥∫Dk,gt1x−t2xdxVAR,Pt,mis≥∑k=1K ∑g=1GkPk,g,thresholdmax=rmin,T.

*(8) Probability of Different-to-Same Mis-Affiliation Induced by Batch-Mode Part: Upper Bound.* Let *r*_max,*T*_ be the radius of the hypersphere corresponding to margin evolving granularity. Assume that auxiliary curves *y* = *t*_1_(*x*) and *y* = *t*_2_(*x*) are constructed under *threshold* = *r*_max,*T*_. On the basis of the previous parts of proof, we can draw the following inequalities:

(5) Pk,g≤∫Dk,gt1x−t2xdxVAR,Pt,mis≤∑k=1K ∑g=1GkPk,g,thresholdmax=rmax,T.

### 3.3. Discussions on Theorem 14

(1) Theorem 14 focuses on different-to-same mis-affiliations other than same-to-different mis-affiliations. Hence we assume that we can neglect the distance between Cur_*k*,*g*_ and envelope curve of the corresponding LBC (part (5) of the proof).

The probability of different-to-same mis-affiliation will be lower if this distance is not negligible. Consequently, inequalities (5) still hold. In this case, inequalities (4) give the lower bound under the worst situation.

(2) For simplification, we assume the envelope curve of GR is a circle (part (4) of the proof).  Theorem 14 still holds even if the envelope curve is of arbitrary shape. The core evolving granularity is determined by dist_*w*,min_ (Definition 9). Larger dist_*w*,min_ always means higher *P*_*t*,mis_ regardless of the envelope curves shape, or vice versa.

(3) Based on Theorem 14, we can declare that *P*_*t*,mis_ is positively related to the hypervolume of GR's envelope body. In the batch-mode part of step *t*, we can partition **X**_*t*_ into more clusters (or micro-clusters) such that data points of each cluster (or micro-cluster) scatter within a smaller range in the *d*-dimensional space.

(4) The batch-mode part of ordinary block-wise algorithm clusters **X**_*t*_ without a global view of ⋃_*t*′=1_^*t*^ **X**_*t*′_. Consequently, **C**_*t*,new_ is facing a high probability of different-to-same mis-affiliations. This means that numerous new clusters (or micro-clusters) in **C**_*t*,new_ are containing heterogeneous data points. No matter what methods the algorithm uses to identify and merge homogeneous clusters (or micro-clusters) between **C**_*t*−1_ and **C**_*t*,new_ (incorporates **C**_*t*,new_ into **C**_*t*−1_ to obtain **C**_*t*_), clusters in **C**_*t*_ will dramatically differentiate from the benchmark clusters up to step *t*.

### 3.4. Theorem of Parallelism

Let incAlgorithm represent an incremental clustering algorithm. Step *t* of incAlgorithm includes two parts: batch-mode part and incremental part. The batch-mode part uses a GPGPU-friendly batch-mode clustering algorithm to partition **X**_*t*_ into micro-clusters. The incremental part merges these micro-clusters into *C*_*t*−1_ sequentially and obtains *C*_*t*_ if *t* ≥ 2. The incremental part clusters the micro-clusters sequentially to obtain *C*_1_ if *t* = 1.

**Theorem 15 .**

Let *Gra*_*ave*,*T*_ represent average evolving granularity of incAlgorithm up to step *T*. Serial Shrink Rate of incAlgorithm is negatively related to *Gra*_*ave*,*T*_.

**Proof**

Suppose incAlgorithm totally clustered *N* data points up to step *T*. Let mClu_*i*_ ∈ ⋃_*t*=1_^*T*^ **C**_*t*,new_  (*i* = 1,2, 3,…, micro*N*_*T*_) be a micro-cluster, where micro*N*_*T*_ = ⋃_*t*=1_^*T*^micro_*t*_ and micor_*t*_ is the number of micro-clusters obtained by batch-mode part of step *t*. Larger Gra_ave,*T*_ means that each cluster in ⋃_*t*=1_^*T*^ **C**_*t*,new_ tends to contain more data points. Thus ⋃_*t*=1_^*T*^micro_*t*_ decreases. Pursuant to the above analysis and Definition 13, SSR declines when Gra_ave,*T*_ increases.

Pursuant to Theorem 15 and Definition 13, parallelism of incAlgorithm is positively related to Gra_ave,*T*_ under measurement of work-depth.

### 3.5. Cause of the Accuracy-Parallelism Dilemma

incAlgorithm degrades to a point-wise incremental clustering algorithm if every micro-cluster produced by the batch-mode part of step *t*  (*t* = 1,2, 3,…) contains and only contains one data point. In this case, Gra_min,*T*_, Gra_max,*T*_, and Gra_ave,*T*_ all reach the lowest bound. No different-to-same mis-affiliation occurs in batch-mode part and the clustering accuracy tends to rise. However, SSR = 1 and parallelism of incAlgorithm reach the lowest bound under measurement of work-depth.

Parallelism of incAlgorithm rises with the growth of Gra_ave,*T*_. Nevertheless, different-to-same mis-affiliation inevitably occurs. Whatever method the incremental part of step *t*  (*t* = 1,2, 3,…) adopts to execute micro-cluster-level clustering, incAlgorithm cannot eliminate such mis-affiliations. Moreover, such mis-affiliations will exert negative influence on the operation of identifying homogeneous micro-clusters. Consequently, clustering accuracy drops. However, SSR decreases and parallelism rises.

## 4. Experiments

In this section, we validate Theorems 14 and 15 through a demo algorithm. Details of this demo algorithm can be found in our previous work [6]. Let demoAlgorithm represent the demo algorithm. The batch-mode part of demoAlgorithm uses mean-shift algorithm to generate micro-clusters. The incremental part of demoAlgorithm extends Self-organized Incremental Neural Network (SOINN) algorithm [7] to execute micro-cluster-level clustering. We validate the variation trends of accuracy and parallelism with respect to evolving granularity. Issues such as improving cluster accuracy of the demo algorithm and adaptively seeking balance between accuracy and parallelism are left as future work.

The benchmark algorithm of demoAlgorithm is batch-mode mean-shift algorithm. Accuracy metrics include the final cluster number, Peak Signal to Noise Ratio (PSNR), and Rand Index. In addition to Serial Shrink Ratio (SSR), we define parallel-friendly rate (PFR) to measure parallelism of demoAlgorithm.

In order to intuitively show the clustering results, images are used as input datasets. The clustering output is the segmented image. Evolving granularity is measured by bandwidth parameters of mean-shift algorithm. Bandwidth parameters are noted in the format of (feature bandwidth, spatial bandwidth).

### 4.1. Additional Performance Metrics

*(1) Accuracy Metrics*. Up to step *T*0, the* final cluster number* of demoAlgorithm is the quantity of clusters in **C**_*T*0_. Homogenous data points tend to fall into different clusters in the final result if the incremental clustering algorithm produces excessively more clusters than the benchmark algorithm, or vice versa. Consequently, demoAlgorithm tends to achieve higher accuracy if demoAlgorithm can resolve a closer final cluster number to that of the benchmark algorithm.

PSNR values of both incremental clustering result and benchmark result are calculated with respect to the original image.

*(2) Parallelism Metrics*. Assume that demoAlgorithm runs on a single-core processor. In step *t* (*t* = 1,2, 3,…), the batch-mode part consumes time *T*_1,*t*_ and generates micro_*t*_ micro-clusters; the incremental part consumes time *T*_2,*t*_. Suppose demoAlgorithm totally clustered *N* data points up to step *T*_0_. Up to step *T*_0_, the* parallel-friendly rate* (PFR) of demoAlgorithm is PFR = ∑_*t*=1_^*T*0^ *T*_1,*t*_/(∑_*t*=1_^*T*0^(*T*_1,*t*_ + *T*_2,*t*_)). Larger PFR means superior parallelism.

### 4.2. Hardware and Software Environment

All experiments are executed on a platform equipped with an Intel Core2TM E7500 CPU, and a NVIDIA GTX 660 GPU. The main memory size of CPU is 4 GB. Linux of 2.6.18-194.el5 kernel is used with gcc 4.1.2 and CUDA5.0.

CUDA grid size is always set to 45. CUDA block size is set to 64. Double precision data type is used in floating point operations on both CPU and GPGPU. Batch-mode mean-shift is used as benchmark algorithm. Uniform kernel is used with mean-shift algorithm [33] for both batch-mode part of demoAlgorithm and benchmark algorithm.

### 4.3. Input Data Sets

We use grayscale images as input. One pixel corresponds to a input data point. The dimension of a data point (vector) is *d* = 3. Components of the 3-dimensional vector are *X*-coordinate of the pixel, *Y*-coordinate of the pixel, and grayscale value of the pixel, respectively. We selected ten natural scenery images (data set 1) and twenty aerial geographical images (data set 2) from USC SIPI data set [34]. Each image is evenly divided into 128 × 128 data blocks. These blocks are successively input to demoAlgorithm. Every image of data set 1 is incrementally clustered in CPU-only mode and GPGPU-powered mode, separately. Incrementally clustering an image of data set 2 in CPU-only mode is excessively time-consuming due to the large data volume. Consequently, the task of incrementally clustering these images is only executed in GPGPU-powered mode.

### 4.4. Experiment Results and Discussion

Bandwidth parameters (8,6) and (10,8) are excessively large for mean-shift algorithm (both incremental part of demoAlgorithm and benchmark algorithm). Under this parameter setting, accuracy of benchmark clustering results is excessively inferior. Consequently, it is not meaningful to compare accuracy between the incremental clustering algorithm and the benchmark. Thus, we omitted the experiment results under granularities (8,6) and (10,8) in Tables 1 and 2 and Figures 4, 5, and 6.

(*1) Data Set 1: Natural Scenery Images*.  Table 1 shows the final cluster numbers resolved by the demo algorithm and the benchmark algorithm. Suppose we single out the cluster number values from column 2 (or 3, 4). Afterwards, we compute the average of this column of values. Let ave*N*_inc_ represent this average. Suppose we single out cluster number values out of column 5 (or 6, 7). Afterwards, we compute the average of this column of values. Let ave*N*_bench_ represent this average. For example, ave*N*_inc_ is (2372 + 2348 + 1388 + 2466 + 1715 + 2208 + 2558 + 2374 + 1631 + 2607)/10 = 2166.7 in terms of column 2. ave*N*_bench_ is (2517 + 2481 + 1469 + 2540 + 1838 + 2326 + 2656 + 2425 + 1700 + 2766)/10 = 2271.8 with regard to column 5.

Under the three ascending granularities, values of ave*N*_inc_/ave*N*_bench_ are 0.9537, 0.9578, and 0.9726, respectively. The final cluster number of demo algorithm is in acceptable agreement with that of the benchmark algorithm.

**Figure 3:**
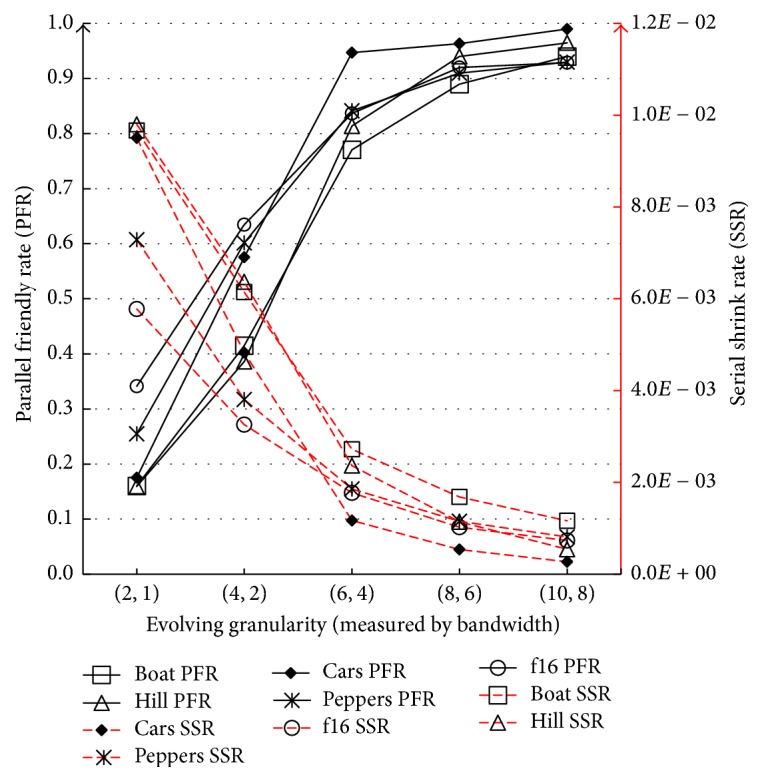
Data set 1: variation trends of PFR and SSR.

**Figure 4:**
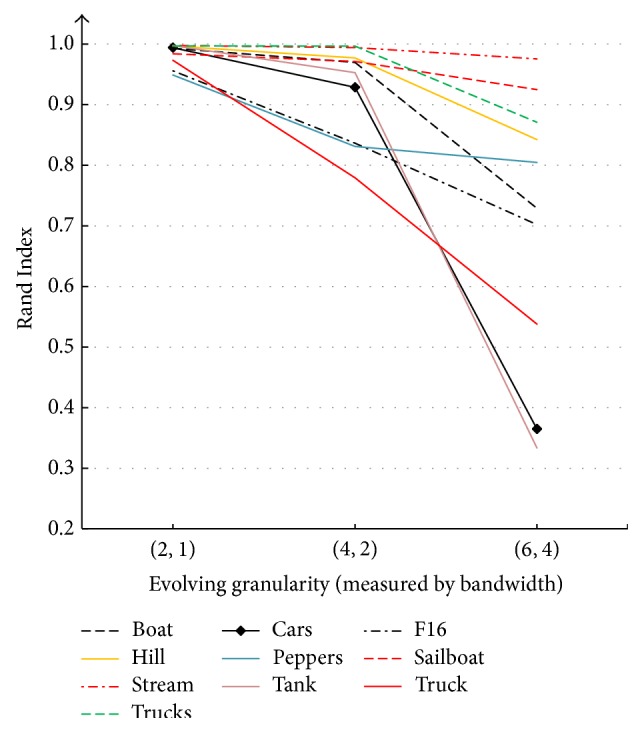
Data set 1: variation trends of Rand Index with respect to evolving granularity.

Figure 3 shows PFR and SSR of the first five inputs from data set 1. The figure reflects positive relation between evolving granularity and parallelism. We omitted the other images due to the fact that they show analogue trends.  Figure 4 illustrates the negative relation between accuracy and evolving granularity.

We select granularity (4,2) as a balance point between accuracy and parallelism based on experience.* truck* has the* lowest* Rand Index value on this balance point.

**Figure 5:**
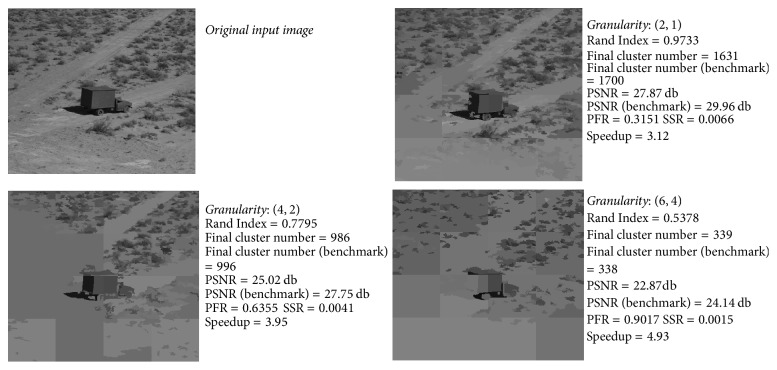
*truck*: original image and incremental clustering results under three ascending granularity values.

Figure 5 shows the original image and experiment results on* truck*. We can draw a similar conclusion from Figure 5 as that of Figures 3 and 4: speedup rises and accuracy degrades when evolving granularity increases. Moreover, Figure 5 shows the final cluster number values and PSNR values of both incremental clustering result and benchmark result, as well as the speedup. In addition to validating Theorems 14 and 15, our demo algorithm can produce acceptable-accuracy result on certain inputs such as* truck*.

(*2) Data Set 2: Aerial Geographical Images*. We continue to use symbols explained in part one of this subsection. Values of (ave*N*_inc_/ave*N*_bench_) under three ascending granularities are 0.9954, 1.1063, and 1.1345, respectively. Overall, the final cluster number of our demo algorithm is close to that of the benchmark algorithm.

In order to avoid unnecessary details, we only list the maximum and minimum Rand Index values (and corresponding image names) under each evolving granularity in Table 2. Similarly, we only list the maximum and minimum SSR values in Table 3. These experiment results can still validate Theorems 14 and 15. We still choose granularity (4,2) as a balance point between accuracy and parallelism based on experience. *usc*.2.02 has the* lowest* Rand Index value on granularity (4,2).

**Figure 6:**
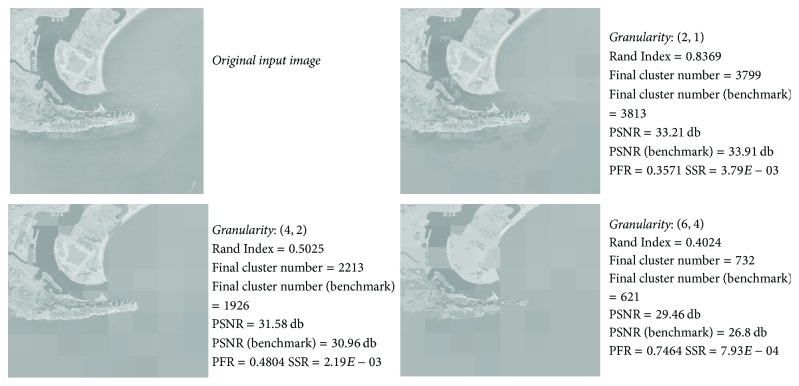
*usc22.02*: original image and incremental clustering results under three ascending granularity values.

Figure 6 shows the final cluster number values and PSNR values of both incremental clustering result and benchmark result. In addition to validating Theorems 14 and 15, our demo algorithm can produce acceptable-accuracy result on certain inputs such as *usc*.2.02.

## 5. Conclusion

In this paper we theoretically analyzed the cause of accuracy-parallelism dilemma with respect to the GPGPU-powered incremental clustering algorithm. Theoretical conclusions were validated by a demo algorithm.

Our future work will focus on identifying the suitable granularity for a given incremental clustering task and decreasing mis-affiliations through variable data-block-size.

## Figures and Tables

**Table 1:** Comparison of final cluster number.

	Demo algorithm			Benchmark algorithm		
	(2,1)	(4,2)	(6,4)	(2,1)	(4,2)	(6,4)
Boat	2372	1444	589	2517	1536	632
Cars	2348	1151	261	2481	1133	270
f16	1388	749	406	1469	827	356
Hill	2466	1552	525	2540	1623	540
Peppers	1715	845	385	1838	943	413
Sailboat	2208	1762	1051	2326	1817	1106
Stream	2558	2312	1524	2656	2437	1574
Tank	2374	1137	172	2425	1146	178
Truck	1631	986	339	1700	996	338
Trucks	2607	2256	681	2766	2360	693

**Table 2:** Dataset 2: max and min Rand Index under ascending granularities.

	Granularity (measured by bandwidth)		
	(2,1)	(4,2)	(6,4)
Max	0.9997	0.9983	0.9850
	(usc2.2.05)	(usc2.2.17)	(usc2.2.17)
Min	0.8369	0.5025	0.1501
	(usc2.2.02)	(usc2.2.02)	(usc2.2.07)

**Table 3:** Dataset 2: max and min SSR values under ascending granularities.

	Granularity (measured by bandwidth)				
	(2,1)	(4,2)	(6,4)	(8,6)	(10,8)
Min	3.7*E* − 03 (usc2.2.02)	2.1*E* − 03 (usc2.2.02)	6.3*E* − 04 (usc2.2.03)	6.1*E* − 04 (usc2.2.03)	1.2*E* − 04 (usc2.2.03)
Max	1.0*E* − 02 (usc2.2.17)	9.5*E* − 03 (usc2.2.17)	6.2*E* − 03 (usc2.2.17)	3.3*E* − 03 (usc2.2.01)	2.1*E* − 03 (usc2.2.08)
